# STAT1-dependent and -independent pulmonary allergic and fibrogenic responses in mice after exposure to tangled versus rod-like multi-walled carbon nanotubes

**DOI:** 10.1186/s12989-017-0207-3

**Published:** 2017-07-17

**Authors:** Katherine S. Duke, Alexia J. Taylor-Just, Mark D. Ihrie, Kelly A. Shipkowski, Elizabeth A. Thompson, Erinn C. Dandley, Gregory N. Parsons, James C. Bonner

**Affiliations:** 1 Toxicology Program, Department of Biological Sciences, North Carolina State University, Campus Box 7633, Raleigh, NC 27695-7633 USA; 2 Department of Chemical and Biomolecular Engineering, North Carolina State University, Raleigh, NC 27695 USA

**Keywords:** Carbon nanotubes, Lung, Fibrosis, Growth factors, Transcription factors

## Abstract

**Background:**

Pulmonary toxicity of multi-walled carbon nanotubes (MWCNTs) is influenced by physicochemical characteristics and genetic susceptibility. We hypothesized that contrasting rigidities of tangled (t) versus rod-like (r) MWCNTs would result in differing immunologic or fibrogenic responses in mice and that these responses would be exaggerated in transgenic mice lacking the signal transducer and activator of transcription-1 (STAT1), a susceptible mouse model of pulmonary fibrosis.

**Methods:**

Male wild type (*Stat1* ^*+/+*^) and STAT1-deficient (*Stat1* ^*−/−*^) mice were exposed to 4 mg/kg tMWCNTs, rMWCNTs, or vehicle alone via oropharyngeal aspiration and evaluated for inflammation at one and 21 days post-exposure via histopathology, differential cell counts, and cytokine levels in bronchoalveolar lavage fluid (BALF). Granuloma formation, mucous cell metaplasia, and airway fibrosis were evaluated by quantitative morphometry. Airway epithelial cell proliferation was assessed by bromodeoxyuridine (BrdU) incorporation. Cytokine protein levels in BALF and serum IgE levels were measured by ELISA. Lung protein Smad2/3 levels and activation were measured by Western blot. Lung mRNAs were measured by PCR.

**Results:**

There was a 7-fold difference in rigidity between tMWCNTs and rMWCNTs as determined by static bending ratio. Both MWCNT types resulted in acute inflammation (neutrophils in BALF) after one-day post-exposure, yet only rMWCNTs resulted in chronic inflammation at 21 days as indicated by neutrophil influx and larger granulomas. Both MWCNTs induced BrdU uptake in airway epithelial cells, with the greatest proliferative response observed in rMWCNT-exposed mice after one-day. Only rMWCNTs induced mucous cell metaplasia, but this index was not different between genotypes. *Stat1* ^*−/−*^ mice had higher levels of baseline serum IgE than *Stat1* ^*+/+*^ mice. Greater airway fibrosis was observed with rMWCNTs compared to tMWCNTs, and exaggerated airway fibrosis was seen in the *Stat1* ^*−/−*^ mouse lungs with rMWCNTs but not tMWCNTs. Increased fibrosis correlated with elevated levels of TGF-β1 protein levels in the BALF of *Stat1* ^*−/−*^ mice exposed to rMWCNTs and increased lung Smad2/3 phosphorylation.

**Conclusions:**

Rigidity plays a key role in the toxicity of MWCNTs and results in increased inflammatory, immunologic, and fibrogenic effects in the lung. STAT1 is an important protective factor in the fibroproliferative response to rMWCNTs, regulating both induced TGF-β1 production and Smad2/3 phosphorylation status. Therefore, both rigidity and genetic susceptibility should be major considerations for risk assessment of MWCNTs.

**Electronic supplementary material:**

The online version of this article (doi:10.1186/s12989-017-0207-3) contains supplementary material, which is available to authorized users.

## Background

Carbon nanotubes (CNTs) are engineered nanomaterials that have structural similarities to asbestos because of their fiber-like shape and biopersistence [1–3]. While little is known about their adverse human health effects, rodent studies show multi-walled (MW) CNTs, like asbestos fibers, possess carcinogenic activity or cause pulmonary fibrosis after inhalation exposure or oropharyngeal aspiration [4–6]. Also reminiscent of asbestos fibers, MWCNTs reach the lung pleura after inhalation exposure in mice, where they irritate the pleural lining and cause pleural inflammation and subpleural fibrosis [7]. While some MWCNTs delivered to the lungs could translocate to other organs after pulmonary exposure, a significant fraction are biopersistent and remain in the lung and pleura up to a year after exposure in rodents, leading to chronic DNA damage and tissue fibrosis [8].

MWCNTs are a heterogeneous class of materials that can mediate different pathogenic responses in the lung, depending on their physicochemical properties. For instance, lung exposure to rod-like (r) MWCNTs can lead to allergic airway inflammation and mucous cell metaplasia, while exposure to tangled (t) MWCNTs causes non-allergic lung inflammation with no mucous cell metaplasia [9–11]. Other properties of MWCNTs have been shown to play a role in their pulmonary toxicity (i.e. length, surface charge, residual metals) yet only a few studies comparing MWCNTs with contrasting rigidity have been conducted [9, 12]. However, to our knowledge there are no studies that address genetic susceptibility to allergic or fibrogenic responses to MWCNTs with different rigidities. Examining the differences in allergic or fibroproliferative responses between tMWCNTs and rMWCNTs in genetically susceptible mouse models may elucidate differences in the fibrotic mechanisms of these MWCNTs.

Signal transducer and activator of transcription-1 (STAT1) is a transcription factor activated primarily by interferons (IFN-α, −β, −γ), growth factors (e.g., epidermal growth factor (EGF), platelet derived growth factor (PDGF)), or by oxidative stress [13–15]. Upon activation, STAT1 is phosphorylated and homodimerizes with another STAT1 molecule, or heterodimerizes with other STAT family members (e.g. STAT2). Homodimers or heterodimers then translocate to the nucleus to bind a DNA consensus sequence; each STAT1 dimer has a high affinity for its specific response element [16]. STAT1 serves as a protective response to injury, and is responsible for activating the transcription of key genes involved in cell viability, growth arrest, apoptosis, and differentiation [17, 18]. *Stat1* ^−/−^ mice exhibit increased fibrosis following exposure to the chemotherapeutic drug bleomycin compared to wild type (*Stat1* ^+/+^) mice, demonstrating that STAT1 is protective against fibrogenesis [19]. Also, ovalbumin sensitized *Stat1* ^−/−^ mice that were subsequently challenged with tMWCNTs by oropharyngeal aspiration exhibit greater airway fibrosis than *Stat1* ^+/+^ mice, indicating that STAT1 plays a role in the immune response to tMWCNTs and allergens [11]. Moreover, fibroblasts isolated from lungs of *Stat1* ^−/−^ mice are more responsive in vitro to exogenous transforming growth factor (TGF)-β1, produce more collagen than *Stat1* ^+/+^ fibroblasts treated with TGF-β1 and display an increased proliferative response in vitro to PDGF or EGF compared to *Stat1* ^+/+^ fibroblasts [19].

It is unknown if STAT1 regulates differential fibrogenic or immune responses to tMWCNTs versus rMWCNTs. In the present study, we hypothesized that MWCNTs with different rigidities would produce different pulmonary immunologic and fibroproliferative responses and that these effects would be enhanced in *Stat1* ^*−/−*^ mice. We investigated MWCNT-induced lung inflammation, allergic airway remodeling and fibrosis in *Stat1* ^+/+^ or *Stat1* ^−/−^ mice in vivo for one and 21 days after oropharyngeal aspiration of tMWCNTs or rMWCNTs. We observed that rMWCNTs caused more persistent lung inflammation, as well as airway mucous cell metaplasia and larger granulomas compared to tMWCNT exposure in wild type *Stat1* ^*+/*+^ mice. Interestingly, compared to *Stat1* ^*+/*+^ mice, *Stat1* ^−/−^ mice exposed to rMWCNTs but not tMWCNTs exhibited enhanced airway fibrosis, airway epithelial cell proliferation, exaggerated serum IgE, increased levels of lung TGF-β1 and increased activation of the TGF-β1-induced transcription factors, Smad2 and Smad3. The results from this study are important because they reveal an interaction between a physicochemical attribute (rigidity) and a genetic factor (STAT1) to further our understanding of susceptibility to pulmonary fibrogenesis and allergic immune responses. Moreover, we identify a novel mechanism of enhanced lung fibrogenesis in *Stat1* ^−/−^ mice induced by rMWCNTs that involves exaggerated TGF-β1 production and Smad2/3 activation.

## Results

### Characterization of MWCNT rigidity

**Fig. 1:**
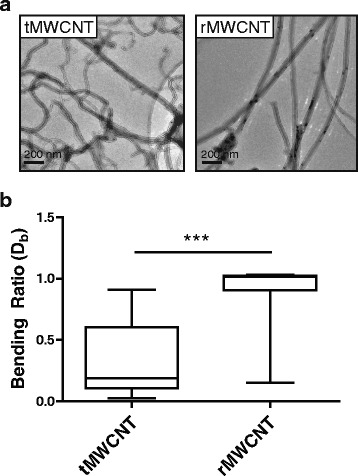
Rigidity measurements of multi-walled carbon nanotubes (MWCNTs). **a** Transmission electron microscope (TEM) images of the tangled (t) MWCNTs and rod-like (r) MWCNTs showing the gross differences in rigidity taken at 10000X. **b** Box-and-whisker plots of the bending ratios (D_b_) of tMWCNTs and rMWCNTs (***p < 0.001 between means of MWCNT D_b_ as determined by *t*-test)

Both tMWCNTs and rMWCNTs have been previously characterized for average residual metal content, length, and width and these data are summarized in Additional file 1 [7, 10]. We further characterized these MWCNTs based on their rigidity using a measure of the bending ratio (D_b_) to approximate the static bending persistence length (SBPL) (i.e. the average length between each bend in the tube) (Fig. 1). TEM images clearly show that tMWCNTs are more tortuous compared to rMWCNTs (Fig. 1a). The rMWCNTs, with a D_b_ of 0.8996 (stdev 0.2743) and a SBPL of 0.860, are about seven-fold more rigid than the tMWCNTs with a D_b_ of 0.162 (stdev 0.3300) and a SBPL of 0.1191 (Fig. 1b).

### Macrophage uptake and inflammation

**Fig. 2:**
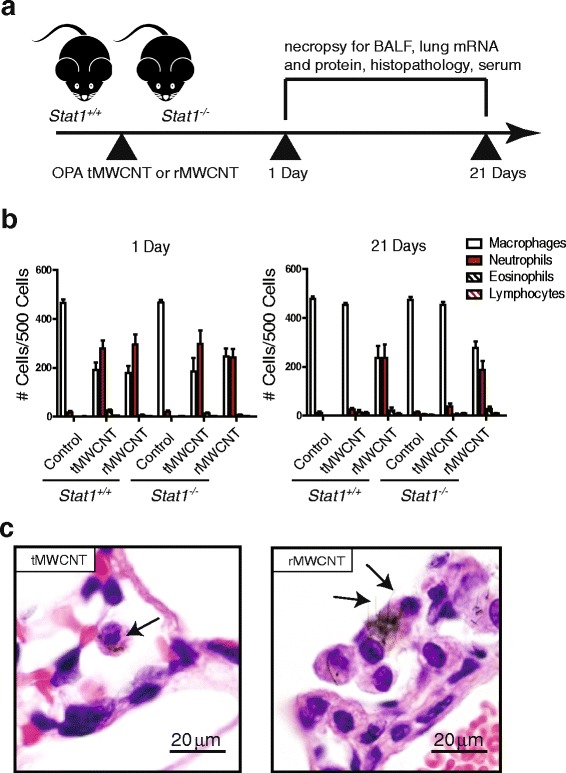
Acute pulmonary response after oropharyngeal aspiration (OPA) to t- or r-MWCNTs. **a** Illustration of experimental design. **b** Differential cell counts quantified from Cytospins of BALF from *Stat1* ^*+/+*^ and *Stat1* ^*−/−*^ mice exposed to tMWCNTs or rMWCNTs. **c** Hematoxylin and eosin-stained (H&E) lung sections from mice treated with tMWCNTs or rMWCNTs at one-day post-exposure showing uptake of tMWCNTs and rMWCNTs by alveolar macrophages (*arrows*). Note frustrated phagocytosis of rMWCNTs but not tMWCNTs by macrophages

*Stat1* ^*+/+*^ and *Stat1* ^*−/−*^ mice exposed to tMWCNTs or rMWCNTs by oropharyngeal aspiration (OPA) were evaluated for changes in bronchoalveolar lavage fluid (BALF) inflammatory cells at one and 21 days post-exposure according the protocol illustrated in Fig. 2a. BALF differential cell counts showed that both *Stat1* ^*+/+*^ and *Stat1* ^*−/−*^ mice exhibited similar increases in infiltrating neutrophils at one-day post-exposure (Fig. 2b). Neutrophilic inflammation in the tMWCNT-treated mouse lungs resolved by 21 days, while neutrophilia in the lungs of rMWCNT-treated mice remained elevated at 21 days in both *Stat1* ^*+/+*^ and *Stat1* ^*−/−*^ mice (Fig. 2b). The tMWCNTs are flexible and were completely engulfed by alveolar macrophages, while many of the rMWCNTs protruded from macrophages, indicating frustrated phagocytosis (Fig. 2c).

### MWCNT-induced granulomatous inflammation

**Fig. 3:**
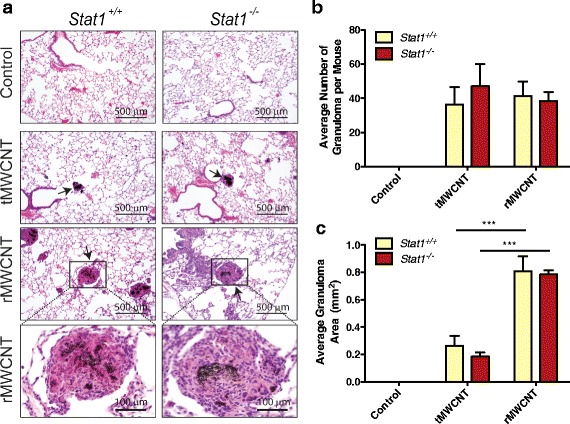
Granuloma development at 21 days post-exposure to tMWCNTs or rMWCNTs. **a** Representative H&E photomicrographs of 21 day control, tMWCNT or rMWCNT-exposed mouse airways showing granulomas (*arrows*) taken at 200X magnification (scale bars equal 500 μm). Insets of rMWCNT granulomas (Scale bars equal 100 μm) taken at 400X magnification. **b** Average number of granulomas per 3 lung sections per mouse. **c** Average size of granulomas measured by quantitative morphometry as described in Methods. (***p < 0.001 between tMWCNTs and rMWCNTs)

To ascertain if the *Stat1* ^*+/+*^ versus *Stat1* ^−/−^ genotypes determine differential chronic pulmonary inflammatory responses to each MWCNT type, the lungs of mice were analyzed for granuloma size and cellularity. Histopathologic analysis of lung tissue from MWCNT-treated mice showed alveolar parenchyma dispersion of MWCNTs and predominantly multifocal granulomatous inflammation and bronchitis (Fig. 3a). The granulomas observed following rMWCNT treatment contained a high number of neutrophils and eosinophils after 21 days. This is notable bearing in mind that classical granulomas consist of epithelioid, multinucleated giant cells, and a very sparse eosinophil or neutrophil presence (Fig. 3a insets). Only rMWCNT exposure resulted in bronchial inflammation and airway epithelial growth over the foreign body protrusions of rMWCNTs into the lumen of the airway, similar to some pathologic features of bronchiolitis obliterans. Quantitative analysis of granuloma number showed that at the same dose of 4 mg/kg, both types of MWCNTs induced similar numbers of granulomas at 21 days with no differences between genotypes (Fig. 3b). While granulomas were observed in both tMWCNT and rMWCNT-treated mice after 21 days, granuloma size was significantly greater in rMWCNT-treated mice compared to tMWCNT-treated mice, although there were no significant differences between *Stat1* ^*+/+*^ and *Stat1* ^−/−^ mice (Fig. 3c). Together, these data demonstrate that rMWCNTs caused a more severe chronic inflammatory state in the lung, resulting in larger granuloma formations and that this inflammatory response was STAT1-independent.

### Airway epithelial cell proliferation after MWCNT exposure

**Fig. 4:**
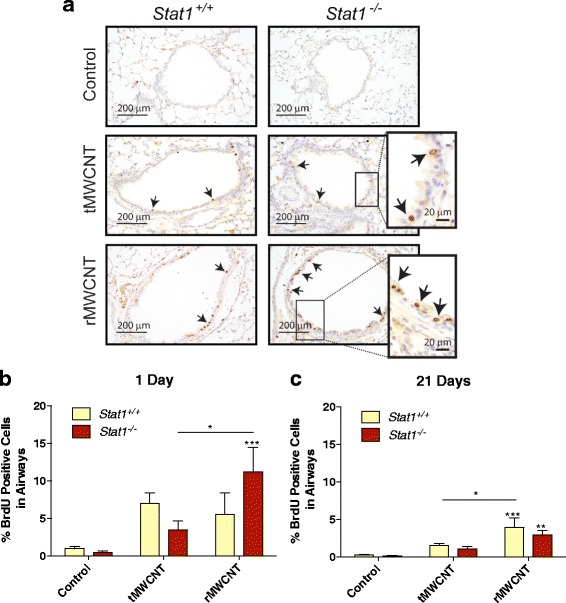
Airway epithelial cell proliferation in *Stat1* ^*+/+*^ and *Stat1* ^*−/−*^ mice after one and 21 days exposure to MWCNTs. **a** Bromodeoxyuridine (BrdU) immunostaining at one-day post-exposure to tMWCNTs or rMWCNTs showing BrdU-positive *brown* nuclei of proliferating cells (*arrows*). **b** Quantification of BrdU positive cells per airway after one and **c** 21 days of exposure. (***p < 0.001 compared to control; *p < 0.05 between tMWCNTs and rMWCNTs)

To determine if tMWCNTs or rMWCNTs caused lung cell proliferation, mice were injected i.p. with BrdU one-hour prior to euthanasia and incorporation of BrdU into nuclei was visualized by immunohistochemistry (Fig. 4a). Vehicle treatment did not cause a significant increase in BrdU uptake in *Stat1* ^*+/+*^ or *Stat1* ^*−/−*^ mice at either one or 21 days (<1% BrdU-positive cells in airways). Both tMWCNTs and rMWCNTs caused a modest and similar increase in BrdU uptake in the airway epithelium of *Stat1* ^*+/+*^ mice at one-day post-exposure (5–10% BrdU-positive cells in airways). In contrast, rMWCNTs caused a significant increase in BrdU uptake in the airway epithelium of *Stat1* ^*−/−*^ mice (10–15% BrdU-positive cells in airways) compared to tMWCNT treatment or vehicle at one-day post-exposure in *Stat1* ^*−/−*^ mouse lungs, but there was no significant difference compared to *Stat1* ^*+/+*^ MWCNT-exposed mouse lungs (Fig. 4b). At 21 days post-exposure BrdU uptake in response to MWCNTs had subsided to ~5% BrdU-positive cells or less, although there was a significant increase in proliferation of the airway epithelial cells in the lungs of both *Stat1* ^*+/+*^ and *Stat1* ^−/−^ mice treated with rMWCNTs compared to tMWCNTs or vehicle control (Fig. 4b).

### MWCNT-induced mucous cell Metaplasia

**Fig. 5:**
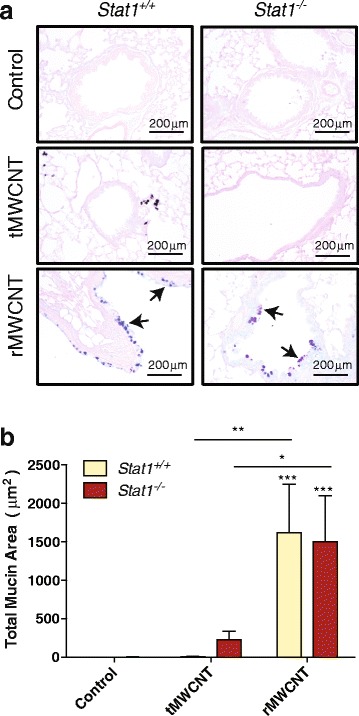
Mucous cell metaplasia after exposure to tMWCNTs or rMWCNTs. **a** Representative photomicrographs of Alcian-*blue* Periodic Acid-Schiff (AB-PAS)-stained lungs from 21 days post-exposure. AB-PAS positive goblet cells are indicated by *arrows*. **b** Quantification of mucous cell metaplasia as average mucous stain per total area of airway. (***p < 0.001 compared to controls; **p < 0.01 or *p < 0.05 between tMWCNTs and rMWCNTs)

We observed airway mucous cell metaplasia in the lungs of both *Stat1* ^*+/+*^ and *Stat1* ^−/−^ mice exposed to rMWCNTs at 21 days but not in mice treated with tMWCNTs (Fig. 5a). Mucous cell metaplasia in response to rMWCNTs was evident by the appearance of AB-PAS-positive goblet cells in the airway epithelium. Quantitative morphometry of all airways sectioned from each mouse revealed a similar increase in AB-PAS goblet cells induced by rMWCNTs in both *Stat1* ^*+/+*^ and *Stat1* ^−/−^ mice (Fig. 5b).

### MWCNT-induced serum IgE and allergic cytokine pulmonary mRNA levels

**Fig. 6:**
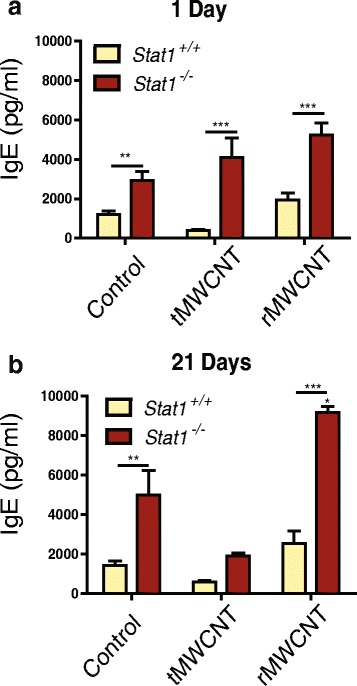
Quantification of immunoglobulin E (IgE) by ELISA in the serum from *Stat1* ^*+/+*^ and *Stat1* ^*−/−*^ mice. **a** Serum IgE concentrations one-day and **b** 21 days post-exposure to tMWCNTs or rMWCNTs. (***p < 0.001 or **p < 0.01 between genotypes, *p < 0.05 compared to control)

There was an increased amount of basal IgE levels in the serum of *Stat1* ^−/−^ mice compared to *Stat1* ^*+/+*^ mice at one and 21 days after exposure to the pluronic vehicle control (Fig. 6). Exposure to tMWCNTs did not increase serum IgE levels in either *Stat1* ^*+/+*^ or *Stat1* ^−/−^ mice at either time point (Fig. 6). In contrast, rMWCNTs significantly increased levels of serum IgE at 21 days after exposure to rMWCNTs in *Stat1* ^−/−^ mice as compared to *Stat1* ^*−/−*^ vehicle control (Fig. 6b). There was a slight increase in lung IL-4 mRNA expression at one-day post-exposure to rMWCNTs in the *Stat1* ^−/−^ mice compared to *Stat1* ^+/+^ mice (Additional file 2A). This difference abated by 21 days post-exposure (Additional file 2B). IL-13 mRNA levels were not significantly changed by exposure to tMWCNTs or rMWCNTs in either genotype at one or 21 days (Additional file 2C and D).

### MWCNT-induced pulmonary fibrosis

**Fig. 7:**
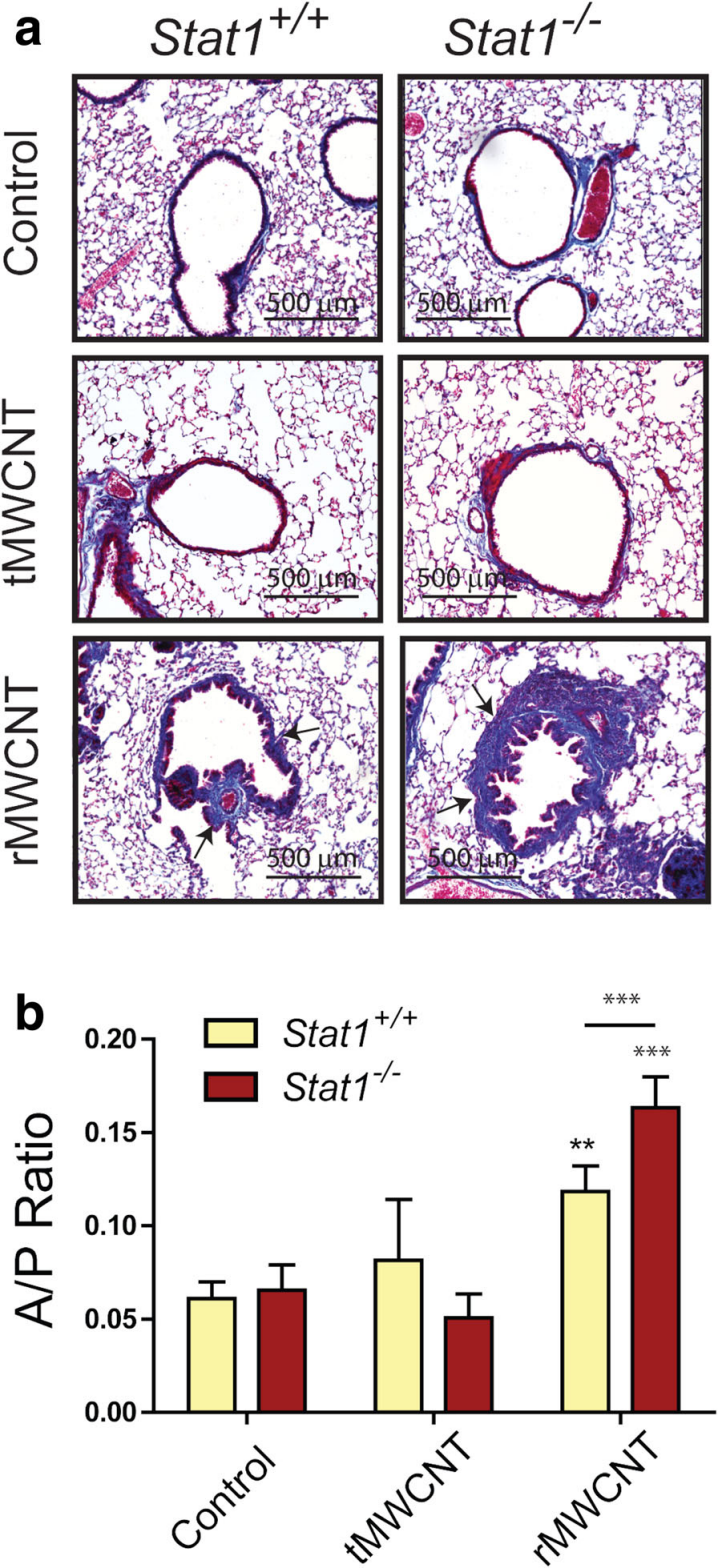
Airway fibrosis in *Stat1* ^*+/+*^ and *Stat1* ^*−/−*^ mice exposed to tMWCNTs or rMWCNTs. **a** Representative photomicrographs taken at 200X magnification of Massons trichrome-stained lung sections showing collagen deposition around airways (*arrows*) in mouse lungs at 21 days post-exposure. Connective tissue (e.g. collagen) stains *blue*. Scale bars equal 500 μm. **b** Area to perimeter (A/P) ratios of mouse lung airway thickness at 21 days post-exposure. (***p < 0.001 or **p < 0.01 compared to control; ***p < 0.001 between tMWCNTs and rMWCNTs)

Lung and airway fibrosis was quantified by morphometric analysis of Masson’s trichrome-stained slides and soluble collagen content from lung protein lysates. Histopathologic evaluation revealed that fibrosis was limited primarily to airways and induced by rMWCNTs but not tMWCNTs. Quantitative morphometry of airway fibrosis to generate area/perimeter ratio measurements indicated significant focal peribronchiolar collagen deposition after exposure to rMWCNTs but not tMWCNTs in the lungs of *Stat1* ^*+/+*^ and *Stat1* ^*−/−*^ mice (Fig. 7a-b). Moreover, *Stat1* ^*−/−*^ mice had significantly more airway fibrosis as compared to *Stat1* ^*+/+*^ mice 21 days after exposure to rMWCNTs (Fig. 7b). Despite the regional increase in airway fibrosis caused by rMWCNTs, there was not a significant difference in total lung collagen at 21 days post-exposure among treatment groups or genotypes as measured by Sircol assay (Additional file 3) or hydroxyproline assay (data not shown).

### MWCNT-induced expression of TGF-β1 and other Profibrogenic cytokines

**Fig. 8:**
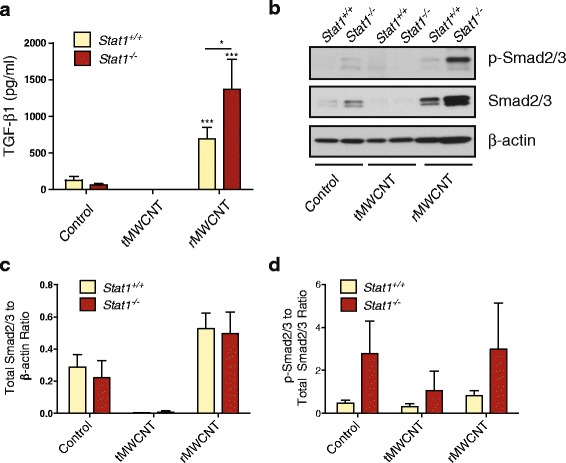
TGF-β1 protein levels and p-Smad2/3 levels in lung tissue from *Stat1* ^*+/+*^ and *Stat1* ^*−/−*^ mice after 21 days of exposure to tMWCNTs or rMWCNTs. **a** TGF-β1 protein in BALF was measured by ELISA (***p < 0.001 compared to controls; *p < 0.05 between genotypes). **b** Representative Western blot showing p-Smad2/3 and total Smad2/3 protein levels in lung tissue. **c** Densitometry of total Smad2/3 levels normalized to β-actin levels. **d** Quantification of the ratio of p-Smad2/3 to total Smad2/3 densitometry levels relative to β-actin

TGF-β1, a primary mediator of fibrosis, was measured at the protein level by ELISA in the BALF of mice. TGF-β1 was significantly increased by rMWCNT-treatment in *Stat1* ^*+/+*^ mouse BALF at 21 days, and further significantly increased in the BALF of *Stat1* ^−/−^ mice that were treated with rMWCNTs (Fig. 8a). In contrast, TGF-β1 was not increased in the BALF from tMWCNT-treated mice at one or 21 days post-exposure (Fig. 8a). TGF-β1 was not detectable in the BALF from any of the treatment groups or genotypes at one-day post-exposure (data not shown). Several other cytokines implicated in fibrogenesis, including osteopontin (OPN), platelet-derived growth factor (PDGF), and interleukin-1β (IL-1β), were also measured at the protein level in BALF or at the mRNA level in lung tissue. The BALF from rMWCNT-exposed mice contained significantly more OPN protein after the one-day exposure, while the BALF from tMWCNT-treated mice contain significantly more OPN 21 days after exposure (Additional file 4A-B). Induction of OPN by MWCNTs was not significantly different between *Stat1* ^*+/+*^ and *Stat1* ^*−/−*^ mice. An ELISA was also conducted to measure the interleukin-1β (IL-1β) in BALF. However, no significant differences in secreted IL-1β protein were observed among treatment groups or genotypes (data not shown). The expression of two PDGF isoforms (PDGF-A and PDGF-B) were measured from lung tissue by Taqman qRT-PCR. While there was a trend for higher levels of PDGF-A and PDGF-B mRNA in the lung tissue from *Stat1* ^−/−^ mice, these increased levels were not significant (Additional file 5).

### Effect of MWCNTs on TGF-β1 signaling molecules

Because changes in TGF-β1 protein levels in BALF closely matched the pathologic changes in airway fibrosis in *Stat1* ^*+/+*^ and *Stat1* ^*−/−*^ mice exposed to rMWCNTs, we sought to investigate components of the TGF-β1 signaling pathway. Total protein lysates were extracted from snap frozen lung tissue and separated by SDS-PAGE followed by Western blot analysis using antibodies specific for TGF-β1 Receptor II (TGF-βRII), phosphorylated Smad2/3 (p-Smad2/3) and total Smad2/3. β-actin was measured as a constitutively expressed protein and used to normalize for densitometry of visualized protein bands (Fig. 8b). The levels of TGF-βRII remained unchanged in both genotypes following either treatment (data not shown). Overall levels of Smad2/3 were increased with rMWCNT exposure and decreased with tMWCNT exposure, but not significantly different among genotypes (Fig. 8c). The ratio of p-Smad2/3 to total Smad2/3 levels displays a higher trend of Smad2/3 activation in *Stat1* ^*−/−*^ mouse lungs compared to *Stat1* ^*+/+*^ with no significant difference between exposures (Fig. 8d). The transcriptional targets of activated p-Smad2/3, Col1a1 and Col1a2, mirror this trend as measured via Taqman qRT-PCR from whole lung mRNA, however the transcription levels of these collagen mRNAs did not change with treatment (Additional file 6).

## Discussion

In this study, we investigated the pulmonary allergic, inflammatory and fibrogenic responses of *Stat1* ^*+/+*^ and *Stat1* ^*−/−*^ mice to rod-like (r) or tangled (t) MWCNTs delivered by oropharyngeal aspiration. Since significant differences were observed in airway fibrogenic responses to rMWCNTs between *Stat1* ^*+/+*^ and *Stat1* ^*−/−*^ mice, we further investigated a mechanism of enhanced fibrosis involving induction of TGF-β1 and Smad2/3 transcription factors that mediate collagen production. Previous studies by other investigators have focused on differential inflammatory, fibrotic, allergic, or carcinogenic effects of r- versus t-MWCNTs in wild type mice [5, 9, 20–22]. However, to our knowledge this is the first time MWCNT rigidity has been quantified and differential allergic or fibrogenic responses to rMWCNTs versus tMWCNTs have been studied in *Stat1* ^*−/−*^ mice, or any other susceptible transgenic mouse strain.

**Fig. 9:**
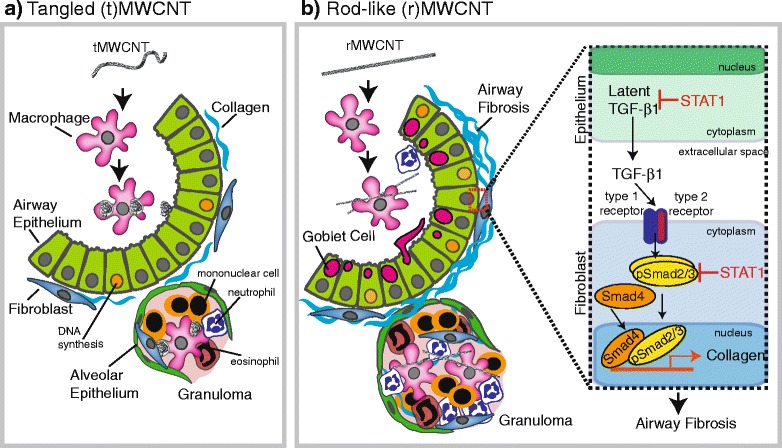
Summary illustration depicting differential chronic lung immune and fibrotic responses at 21 days post-exposure to **a** tangled (t)MWCNTs or **b** Rod-like (r)MWCNTs. The inset in panel B highlights the postulated role of STAT1 in suppressing TGF-β1 production by epithelial cells and Smad2/3 activation levels in fibroblasts

We previously reported that *Stat1* ^*−/−*^ mice are susceptible to lung fibrosis caused by oropharyngeal aspiration of bleomycin or by co-exposure to MWCNTs and ovalbumin allergen [11, 19]. In the latter study, tMWCNTs in the absence of allergen did not cause significant pulmonary fibrosis in *Stat1* ^−/−^ or *Stat1* ^+/+^ mice, an observation confirmed in the current study. In the present study, we observed that rMWCNTs but not tMWCNTs significantly increased airway fibrosis in the lungs of *Stat1* ^+/+^ mice and fibrosis was further increased in *Stat1* ^−/−^ mice focally around airways. Similarly, rMWCNTs but not tMWCNTs significantly increased TGF-β1 levels in the BALF of *Stat1* ^*+/+*^ mice and further increased TGF-β1 in *Stat1* ^*−/−*^ mice after 21 days. However, the increase in TGF-β1 did not correlate with increased total lung collagen mRNAs (Col1a1 and Col1a2) at one or 21 days post-exposure, indicating that a regional increase in airway fibrosis might not be detectable by assays that measure total lung collagen mRNA or protein levels (i.e., Sircol and hydroxyproline assays). In addition, rMWCNTs but not tMWCNTs induced total lung levels of Smad2/3 and greater Smad2/3 phosphorylation was observed in *Stat1* ^*−/−*^ mouse lungs compared to *Stat1* ^*+/+*^ mouse lungs. Therefore, our findings suggest that STAT1 regulates fibrosis through suppressing TGF-β1 production and decreasing Smad2/3 phosphorylation status. No changes were observed in TGF-βRII protein levels in the lungs of *Stat1* ^*+/+*^ or *Stat1* ^*−/−*^ mice treated with or without MWCNTs. These findings suggest that increased airway fibrosis in the lungs of *Stat1* ^*−/−*^ mice exposed to rMWCNTs is mediated by increased levels of TGF-β1 in the BALF as well as increased Smad2/3 activation in lung tissue. A proposed mechanism illustrating the role of STAT1 in suppressing TGF-β1 and Smad2/3 activation in rMWCNT-induced airway fibrosis is shown in Fig. 9.

In the present study, we showed that rMWCNTs or tMWCNTs caused similar acute neutrophilic inflammation in the lungs of either *Stat1* ^*−/−*^ or *Stat1* ^*+/+*^ mice at one-day post-exposure, although only rMWCNTs caused elevated neutrophil counts in the BALF of mice at 21 days (Fig. 2) along with pulmonary fibrosis at 21 days (Fig. 7). The similar acute inflammatory response seen with rMWCNTs and tMWCNTs with fibrosis caused only by rMWCNTs was not surprising as previous studies have shown that acute inflammation is not a prerequisite for fibrogenesis. For example, the glucocorticoid dexamethasone dramatically reduces acute silica-induced lung inflammation in NMRI mice but did not reduce silica-induced fibrosis in these mice, indicating that the development of silica-induced lung fibrosis was uncoupled from acute inflammatory lung responses [23]. In another study, MWCNT-induced acute neutrophilic lung inflammation was ablated in IL-1 receptor knock-out (IL-1R^−/−^) mice, although IL-1R^−/−^ mice developed significantly greater MWCNT-induced lung fibrosis compared to wild type mice [24]. Therefore, our findings indicating that acute inflammation is not a prerequisite for fibrosis is consistent with other reports of particle-induced fibrosis. However, it is possible that chronic sustained neutrophilic inflammation seen in our study could play a role in modifying the fibrotic response to MWCNTs.

The susceptibility of *Stat1* ^−/−^ mice to pulmonary fibrosis is consistent with the well-established function of STAT1 as a primary growth inhibitory signaling pathway for interferons [17, 18]. For example, STAT1 specifically interacts with cyclin D1 and CDK4 to mediate cell cycle arrest in a human fibrosarcoma cell line after treatment with interferon (IFN)-γ [25]. We originally reported that *Stat1* ^−/−^ mouse lung fibroblasts (MLFs) have enhanced proliferative responses to PDGF or EGF in vitro when co-treated with IFN-γ, whereas the proliferative response to these growth factors was inhibited by IFN-γ in *Stat1* ^+/+^ MLFs [19]. The enhanced proliferative response of *Stat1* ^*−/−*^ MLFs was likely due to the fact that IFN-γ activates other pathways independent of STAT1 (e.g., mitogen activated protein kinases (MAPKs) and protein kinase B (Akt)) [17]. Therefore, in the absence of STAT1, IFN-γ signaling can participate in growth promoting pathways otherwise kept in check by the presence of STAT1.

In addition to suppression of fibroblast growth, another anti-fibrotic mechanism of STAT1 involves inhibition of TGF-β1 signaling. We previously established that *Stat1* ^−/−^ MLFs express higher levels of collagen mRNAs and produce more collagen protein than *Stat1* ^+/+^ MLFs when stimulated with TGF-β1 [11]. STAT1 has also been shown to be a negative regulator of hepatic fibrosis, where IFN-γ treatment of *Stat1* ^*+/+*^ mice did not result in fibrosis while the same treatment of *Stat1* ^−/−^ mice resulted in increased hepatic fibrosis and accelerated TGF-β1/Smad signaling [26]. Here, we showed for the first time that *Stat1* ^−/−^ mice have exaggerated levels of TGF-β1 in the bronchoalveolar lavage fluid relative to *Stat1* ^+/+^ mice after exposure to rMWCNTs, demonstrating that STAT1 negatively regulates TGF-β1 production and that rMWCNTs are more potent inducers of TGF-β1 compared to tMWCNTs. We also established that Smad2/3 phosphorylation status was increased in *Stat1* ^−/−^ mice compared to *Stat1* ^+/+^ mice. Collectively, these data suggest that STAT1 suppresses rMWCNT-induced fibrosis by suppressing TGF-β1 production and intracellular Smad2/3 activation (Fig. 9). The mechanism of enhanced phosphorylation of Smad2/3 in *Stat1* ^−/−^ MLFs by rMWCNTs remains to be elucidated. However, others have shown that activated STAT1 homodimers upregulate SOCS1 and Smad7, thereby inhibiting TGF-β1-induced Smad2/3 activation [27, 28]. Also, TGF-β1 suppresses IFN-γ-induced STAT1-dependent gene transcription in epithelial cells through enhancement of protein inhibitor of activated STAT1 (PIAS1) [29]. The opposing roles of TGF-β1 and IFN-γ should be a topic of further investigation in the context of *Stat1* ^*−/−*^ susceptibility to rMWCNTs.

Others have shown that the same rMWCNTs used in the present study increase TGF-β1 in fibrotic foci within the lungs of mice exposed by oropharyngeal aspiration and TGF-β receptor-1 (TGF-βRI) has been implicated as a major regulator of MWCNT-induced fibrogenesis in mice exposed to rMWCNTs [30]. For example, rMWCNTs induced TGF-βRI in human lung fibroblasts in vitro and knock down or inhibition of TGF-βR1 or knock down of Smad2 resulted in decreased collagen production [22, 30]. We were unable to detect TGF-βRI in the present study, but observed that TGF-βRII levels were not changed by rMWCNT treatment or STAT1 deficiency. Nevertheless, it is possible that STAT1 could regulate levels of TGF-βRI. In addition to the collagen synthetic pathways induced by TGF-β1, it is also possible that inhibition of collagen-degrading proteases may be involved in the fibrogenic response to rMWCNTs.

Fibrogenesis stimulated by a foreign body insult (e.g., particles and fibers) is thought to be initiated at least in part by acute injury to epithelial cells. Increased epithelial proliferation marks an injury event where DNA synthesis and cell cycle progression are initiated as a homeostatic repair response, much like what is seen with chrysotile asbestos inhalation exposure [31]. In this study, we observed a significant increase in the proliferation of airway epithelial cells by BrdU uptake after exposure to rMWCNTs (Fig. 4). BrdU uptake at one-day was also induced by tMWCNTs but not to a significant extent. Furthermore, we observed that *Stat1* ^−/−^ mice exhibited significantly greater airway epithelial proliferation compared to vehicle-treated or tMWCNT-treated *Stat1* ^−/−^ mice. BrdU uptake in the airway epithelium of rMWCNT-treated *Stat1* ^*−/−*^ mice was twice that seen in rMWCNT-treated *Stat1* ^*+/+*^ mice, yet this difference was not statistically significant (Fig. 4). The increase in airway epithelial cell BrdU uptake could be mediated by growth factors (e.g., PDGF, EGF) produced by epithelial cells, macrophages, or other pulmonary cell types. We previously reported that primary *Stat1* ^−/−^ MLFs have enhanced proliferative responses to PDGF or EGF in vitro [19]. Therefore, it is possible that airway epithelial cells from *Stat1* ^*−/−*^ mice might have enhanced proliferative responses to growth factors in vivo.

We observed that both rMWCNTs and tMWCNTs produced granulomas, although rMWCNTs produced significantly larger granulomas than tMWCNTs (Fig. 3). Other studies have documented granuloma formation after MWCNT exposure [4–6, 20, 29, 32–34]. Granulomas centrally consist of epithelioid and multinucleated giant macrophages surrounded by activated lymphocytes, typically activated CD4+ T cells [35]. Granulomas resulting from MWCNT treatment have been found to contain CD3+ monocytes and CD4+ T cells, as well as increased levels of osteopontin (OPN) in the granulomatous foci in mouse lungs [36]. In our study, granuloma formation was generally independent of STAT1 as both *Stat1* ^*−/−*^ and *Stat1* ^*+/+*^ mice had a mean granuloma size that was not statistically different between genotypes. Moreover, OPN levels were similar between genotypes, albeit strongly induced by rMWCNTs (Additional file 4). However, two out of six *Stat1* ^*−/−*^ mice exposed to rMWCNTs had at least one exaggerated granuloma, although these “super-granulomas” were rare and did not contribute significantly to differences in granuloma size between genotypes (data not shown). Other investigators have reported that *Stat1* ^−/−^ mice infected with *M. tuberculosis* have larger granuloma development compared their infected *Stat1* ^+/+^ counterparts [37].

Mucociliary clearance is an innate immune response that removes inhaled particles and fibers from the lungs, yet excessive mucous cell metaplasia and mucus hypersecretion contributes to airway obstruction. Mucous cell metaplasia has been observed previously in mice exposed to rMWCNTs [9, 10]. Induction of Th2 cytokines such as IL-4 and IL-13, activation of STAT6, and transcription of mucin genes (*muc5ac* and *muc5b*) contribute to mucin formation in airway epithelial cells [38]. We did not observe significant differences in IL-4 or IL-13 mRNA levels between genotypes or treatment groups, although there was a trend for increased IL-4 mRNA in the lungs of *Stat1* ^*−/−*^ mice treated with rMWCNTs (Additional file 2). However, others have shown that rMWCNTs cause increased IL-13 mRNA levels and allergic airway inflammation in female mice after inhalation exposure [9]. In addition, Hussain et al. observed mucous cell metaplasia after 21 days post-exposure to tMWCNTs in female mice [39]. This contrasts to most other studies performed in male mice. Therefore, sex differences could explain the difference in mechanism of mucous cell metaplasia observed in this study. It is also conceivable that an IL-13-independent mechanism could mediate mucous cell metaplasia in response to rMWCNTs. For example, STAT6 activation can also occur through an alternative mechanism involving STING and TBK1 activation [40].

STAT1 is an IFN-γ activated transcription factor driving the differentiation of naïve T cells to become Th1 cells. In the absence of a Th1 environment, there will more likely be a shift towards a Th2 response. In the present study, induction of a systemic Th2 immune response in *Stat1* ^−/−^ mice was evident by increased levels of serum IgE. Serum IgE levels were especially high in *Stat1* ^*−/−*^ mouse serum and increased greatly following treatment of *Stat1* ^−/−^ mice with rMWCNTs after 21 days (Fig. 6b). However, both *Stat1* ^+/+^ and *Stat1* ^−/−^ mice exhibited increased mucous cell metaplasia with rMWCNT treatment but not tMWCNTs. The reason for a differential systemic allergic response yet similar allergic lung responses between *Stat1* ^*+/+*^ and *Stat1* ^*−/−*^ remains to be elucidated.

In addition to genetic susceptibility, our study highlights the importance of nanotube rigidity. Physicochemical characteristics other than rigidity could also influence the allergic immune and fibrotic potential of MWCNTs. For example, length is an important determinant as intratracheal instillation of long, but not short, MWCNTs causes the formation of granulomas, up-regulation of ECM protease inhibitors, increased collagens, and TGF-β1 production in rats [21]. Thicker and longer MWCNTs induce the greatest DNA damage and induce transcriptional markers of fibrosis compared to thinner, shorter MWCNTs [34, 35]. Finally, differences in residual metal catalysts (Fe in rMWCNTs and Ni in tMWCNTs) could influence toxicity and disease outcome. Collectively, these studies along with our observations in the present work emphasize that multiple physicochemical characteristics should be considered in the design of MWCNTs to reduce or prevent future disease.

## Conclusions

In summary, MWCNT rigidity plays a substantial role in pulmonary toxicity. Moreover, STAT1 is an important protective factor and plays a role in suppressing the fibrogenic response to rMWCNTs by inhibiting TGF-β1 production, intracellular Smad2/3 phosphorylation, and collagen synthesis. Both rigidity and genetic susceptibility should be major considerations for risk assessment and development of MWCNTs.

## Methods

### MWCNT materials and preparation

tMWCNTs were obtained from Helix Material Solutions Inc. (Richardson, TX). rMWCNTs (XRNI MWNT-7 05072001 K28) were obtained from Dale Porter at NIOSH and manufactured by Mitsui & Co (Tokyo, Japan). Pluronic F-68 Solution (#P5556) from Sigma (Saint Louis, MO) was diluted with DPBS to 0.1% and used to bring MWCNTs to a working concentration of 2 mg/mL. MWCNTs were sonicated in a cup horn sonicator for 2 min and were also vortexed vigorously immediately prior to dosing mice 4 mg/kg via oropharyngeal aspiration.

### Rigidity measurements

Measurements were taken as described in Method 3 by the International Standards Organization 11,888:2011 [41, 42]. Ten transmission electron microscope (TEM) images of tMWCNTs and ten of rMWCNTs taken at 10000X were used to perform length measurements of each MWCNT directly from end to end (R) and along the axis (*L*) of each tube per image; these lengths were measured using the Adobe Photoshop C5S ruler tool to convert pixels to μm and trace the imaged MWCNTs. These measurements were averaged into a bending ratio (D_b_) between the mean-squared end-to-end distance <R^2^> and squared contour length (*L* ^2^) to determine the approximate static bending persistence length (SBPL) using the equations:

$$ {\mathrm{D}}_{\mathrm{b}}=<{\mathrm{R}}^2>/{L}^2 $$

$$ \mathrm{SBPL}=\left({{\mathrm{D}}_{\mathrm{b}}}^{\ast } L\right)/2. $$

### Animal care

Pathogen free 6–8 week old adult SV129 male *Stat1* ^+/+^ and *Stat1* ^−/−^ mice were purchased from Taconic Laboratories (Germantown, NY) and housed in an IACUC approved and AALAC-accredited animal facility. Animals were acclimated for two weeks prior to treatments. Animals were housed 1–5 per cage and fed water and LabDiet 5001 rodent diet *ad libitum*.

### Experimental design and collection of mouse samples

The experimental design is illustrated in Fig. 2a. *Stat1* ^*+/+*^ and *Stat1* ^*−/−*^ mice were divided into 3 treatment groups (vehicle, tMWCNT, or rMWCNT) for one and 21 day sample collections. Mice were anesthetized with isoflurane and dosed with 4 mg/kg tMWCNTs (*n* = 6), 4 mg/kg rMWCNTs (*n* = 12), or equal volume pluronic vehicle (*n* = 18) via oropharyngeal aspiration. Half the mice from each treatment group were euthanized via intraperitoneal (i.p.) injection of pentobarbital (Vortech Pharmaceuticals, LTD, Dearborn, MI #NDC 0298–9373-68) at one-day post-exposure and the rest at 21 days post-exposure. Serum was collected immediately and extracted from clotting factors using a BD (Franklin Lakes, NJ) microtainer SST. Two 0.5 mL aliquots of phosphate buffered saline (PBS) were instilled via intratracheal cannulation and retrieved to collect bronchoalveolar lavage fluid (BALF) for cytokine and cellular content. The left lobe of the lung was inflated and fixed for histology with neutral buffered formalin (Azer Scientific, Morgantown, PA #NBF-4-G) and the right lobes were divided equally into RNAlater (Sigma #R0901) for mRNA or snap frozen in liquid nitrogen for protein analysis. Small intestine, heart, spleen, brain, and liver samples were also collected for histology and mRNA analysis.

### Enzyme-linked immunosorbent assay (ELISA)

BALF was analyzed using DuoSet ELISA kits (R & D Systems, Inc., Minneapolis, MN) specific for mouse TGF-β1 (DY1679), Osteopontin (OPN) (DY441), and IL-1β (DY401). Serum IgE was assayed using a BD Pharmingen ELISA kit (557,079, San Jose, CA). Sample concentrations in BALF or serum were derived from absorbance values and converted to concentration values based on standards provided with each kit.

### Cell counts

Differential cell counts were obtained by Cytospin centrifugation of 100 μL of BALF using a Single Cytology Funnel (Fisherbrand, Pittsburgh, PA, 10–354) onto a slide and once dry, stained with DiffQuik (Siemens, Munich, Germany, B4132-1A). Differential cell counts were taken by counting 500 cells per slide/animal to identify relative percentages of macrophages, neutrophils, eosinophils, and lymphocytes.

### Quantitative morphometry of lung granulomas

Granulomas in lung sections stained with Masson’s Trichrome were analyzed by light microscopy (Olympus BX40 microscope). A granuloma was defined as a multi-cellular focal formation consisting of monocytes, macrophages, fibroblasts, and epithelial cells surrounding a MWCNT aggregate. Some granulomas also contained neutrophils and eosinophils. Photomicroscopic images were analyzed using Adobe Photoshop CS5 to determine the area of each granuloma formation by using the lasso tool and converting pixels to μm.

### Airway area to perimeter ratio

An area to perimeter (AP) ratio method was used to quantitatively assess airway fibrosis as previously described [7, 11]. Briefly, light microscopic images of Masson’s trichrome-stained airways were analyzed using Adobe Photoshop CS5; the lasso tool was used to outline the inside perimeter of the airway along the basal membrane of the airway epithelium and along the outside perimeter of the ‘blue-stained’ connective tissue surrounding the airway. The ratio of this inner to outer measurement circumference measurement is referred to as the area to perimeter ratio.

### Bromodeoxyuridine (BrdU) immunohistochemistry (IHC)

Mice received an i.p. injection of 100 mg/kg BrdU in PBS one-hour prior to euthanasia with an i.p. injection of pentobarbital. Formalin-fixed, paraffin embedded blocks of lung or small intestine (positive control) were cut 5 μm with a microtome and mounted on a positively charged slide and dried overnight. The sections were immunostained with anti-BrdU Pure (BD #347580) followed by the Vectastain ABC kit (VectorLabs, Burlingame, CA, #PK-6102) and DAB buffer (BioGenex, San Ramon, CA, #HK542-XAK) as described per manufacturer protocol. BrdU-positive brown-stained nuclei stand out from the hematoxalin counterstain. Photomicroscopic images were analyzed in Adobe Photoshop CS5 using the count tool to measure numbers of BrdU-positive epithelial cells associated with each airway. BrdU-positive cells were normalized for the total number of airway epithelial cells in each airway section. Data are the average percent of BrdU positive cells per airway per mouse in each treatment group.

### Mucin quantification

Photomicroscopic images of Alcian Blue –Periodic Acid Schiff (AB-PAS) stained lung sections were analyzed using Image J software and the area of positive stained blue/purple mucin was quantified as previously described [11].

### RT-PCR

Applied Biosystems high capacity cDNA reverse transcription kit (Fisher #4368814) was used to create cDNA from the mRNA isolated from the right lung lobes using Quick-RNA™ MiniPrep (Zymo Research, Irvine, CA, #R1058) according to the manufacturer’s instructions. The FastStart Universal Probe Master (Rox) (Roche, Basel, Switzerland, #16881300) was then used to run Taqman qPCR on the Applied Biosystems OneStepPlus™ Real-Time PCR System Thermal Cycling Block (ABI, Foster City, CA, Cat#4376598) to determine the comparative C_T_ (ΔΔC_T_) fold change expression of IL-4, IL-13, PDGF-A, PDGF-B, Col1a1, and Col1a2 normalized for β2-microglobulin (B2M) as the endogenous control.

### Collagen analysis

Snap frozen right cranial lung lobes were thawed and 10–50 mg of tissue was prepared for the Sircol Assay (Biocolor Ltd., Carrickfergus, UK, #S1000). The tissues and samples were analyzed according to the manufacturer’s instructions. Briefly, samples were sonicated for 6 min and then treated with Triton-X (Sigma #T8787) overnight. These samples were combined with Sirius Red dye and collagen hydroxyproline residues pelleted by centrifugation at full speed. The pellet was then washed twice with 99.9% cold denatured alcohol before resuspension in an alkali reagent. Absorbance was measured at 540 nm on a microplate reader to determine the concentration of collagen per lung. Samples were assayed in duplicate and collagen content was normalized to protein concentration of lung lysate and reported as μg of soluble collagen per mg of total protein. Collagen content in the lungs of mice was also measured by hydroxyproline assay according to the manufacturers’ instructions (Sigma, St. Louis, MO).

### Immunoblotting

Whole lung protein lysates were isolated from snap-frozen mouse left lung lobes and concentrations determined using the Pierce BCA Protein Assay Kit (ThermoFisher Scientific, Waltham, MA, #23225). Samples were diluted, loaded onto a Novex™ 4–12% SDS-PAGE gel (Invitrogen, Carlsbad, CA, #XP04122BOX), and separated by electrophoresis as described previously [43]. Briefly, samples were transferred to PVDF membranes, blocked, and incubated in primary antibody (1:1000 dilution). Rabbit polyclonal pSmad2/3 (#8828), total Smad2/3, and β-actin (#4967) primary antibodies as well as anti-rabbit (#7074) secondary antibody were purchased from Cell Signaling Technology (Beverly, MA). Following primary antibody incubation, the membranes were washed and then incubated with horseradish peroxidase-conjugated secondary antibody (1:2500 dilution). Enhanced chemiluminescence (ECL) (ThermoFisher Scientific #50–904-9326) was used to visualize immunoblot signals. Protein lysate was extracted from lung tissue of *Stat1* ^*+/+*^ mouse lungs after 21 day exposure to vehicle (*n* = 9), tMWCNT (*n* = 3), and rMWCNT (*n* = 6) and *Stat1* ^*−/−*^ mouse lungs exposed to vehicle (*n* = 9), tMWCNT (*n* = 3), and rMWCNT (*n* = 6). To quantify all immunoblot signals, densitometry was performed as described previously using Adobe Photoshop CS5 [44].

### Statistics

Statistical analysis of the data was performed using GraphPad Prism version 5.0 (GraphPad Software Inc.). A one-way ANOVA with a post hoc Tukey test was used to determine significance between samples. A two-way ANOVA was used with a Bonferroni post-test to compare between genotypes.

## Additional files

Additional file 1: Physicochemical parameters of tangled (t) and rigid (r) multi-walled carbon nanotubes (MWCNTs). (PDF 31 kb)

Additional file 2: Interleukin-4 (IL-4) and IL-13 mRNA expression in *Stat1* ^*+/+*^ and *Stat1* ^*−/−*^ mouse lungs after exposure to tMWCNTs or rMWCNTs. A) Fold change in IL-4 mRNA at one and B) 21 days post-exposure. C) Fold change in IL-13 mRNA expression at one and D) 21 days post-exposure. Expression of mRNA normalized to β2-microglobulin (B2M). (PDF 352 kb)

Additional file 3: Soluble collagen content measured from mouse lungs 21 days post-exposure. Average soluble collagen concentration per lung in each respective treatment after 21 days of exposure to control, tMWCNTs, or rMWCNTs normalized to protein content of sample. (PDF 314 kb)

Additional file 4: Osteopontin (OPN) protein levels in lungs from *Stat1* ^*+/+*^ and *Stat1* ^*−/−*^ mice after one and 21 days of exposure to tMWCNTs or rMWCNTs. A) OPN protein in BALF after one and B) 21 days exposure to vehicle, tMWCNTs, or rMWCNTs as measured by ELISA. (*p < 0.05 or ***p < 0.001 compared to control). (PDF 329 kb)

Additional file 5: Platelet derived growth factor (PDGF) -A and -B expression in *Stat1* ^*+/+*^ and *Stat1* ^*−/−*^ mouse lungs after exposure to tMWCNTs or rMWCNTs. A) Fold change in PDGF-A mRNA at one and B) 21 days post-exposure. C) Fold change in PDGF-B mRNA expression at one and D) 21 days post-exposure. Expression of mRNA normalized to B2M. (PDF 356 kb)

Additional file 6: Expression levels of collagen mRNAs determined via Taqman qRT-PCR of RNA isolated from mouse lungs 21 days post-exposure. A) Fold change in Col1a1 and B) Col1a2 mRNA expression after 21 days post-exposure. Expression of mRNA levels normalized to B2M. (PDF 137 kb)

## Abbreviations

BALF: Bronchoalveolar lavage fluid
BrdU: Bromodeoxyuridine
CNTs: Carbon nanotubes
Db: Static bending ratio
ECM: Extracellular matrix
EGF: Endothelial growth factor
ELISA: Enzyme linked immunosorbent assay
ERK: Extracellular signal related kinase
IFN: Interferon
IgE: Immunoglobulin E
IL: Interleukin
MAD: Mothers against decapentaplegic protein (*Drosophilia*)
MAPK: Mitogen activated protein kinase
MWCNTs: Multi-walled CNTs
OPA: Oropharyngeal aspiration
OPN: Osteopontin
PDGF-A: Platelet derived growth factor-A
PDGF-B: Platelet derived growth factor-B
rMWCNT: Rod-like MWCNT
SBPL: Static bending persistence length.
SMA: Small body size protein (*Caenorhabditis elegans*)
STAT1: Signal transducer and activator of transcription-1
*Stat1*^*−/−*^: STAT1 knockout
*Stat1*^*+/+*^: Wild type for STAT1
TEM: Transmission electron microscope
TGF- βRI: TGF-β receptor I
TGF-β1: Transforming growth factor-β1
TGF-βRII: TGF-β receptor II
Th1: type 1 T helper cell
Th2: type 2 T helper cell
tMWCNT: Tangled MWCNT
