# Supplementary material for: STAT1-dependent and -independent pulmonary allergic and fibrogenic responses in mice after exposure to tangled versus rod-like multi-walled carbon nanotubes
Source: Part Fibre Toxicol. 2017 Jul 17;14:26. doi: 10.1186/s12989-017-0207-3 (PMC5512939; doi:10.1186/s12989-017-0207-3)
Supplement: Supplementary file 1 — Physicochemical parameters of tangled (t) and rigid (r) multi-walled carbon nanotubes (MWCNTs). (PDF 31 kb) [file 12989_2017_207_MOESM1_ESM.pdf]

Additional File 1

Physicochemical parameters of tangled (t) and rigid(r) multi-walled carbon nanotubes (MWCNTs).

MWCNT	Manufacturer	Bending ratio (D_b)	Catalyst	Trace catalyst	Length	Width
tMWCNT	Helix Material Solutions Inc., Richardson, TX	0.162620909	Nickel	5.3%[a]	0.3-50 µm[a]	30-50 nm[a]
rMWCNT	Mitsui & Co, Ltd., Tokyo Japan	0.899625693	Iron	0.32%[b]	3.86 µm[b]	49 ±13.4 nm[b]

[a] Ryman-Rassmussen et al., 2009 (Ref. 7)

[b] Porter *et al.,* 2010 (Ref.10)
