# Supplementary figures and images for: STAT1-dependent and -independent pulmonary allergic and fibrogenic responses in mice after exposure to tangled versus rod-like multi-walled carbon nanotubes
Source: Part Fibre Toxicol. 2017 Jul 17;14:26. doi: 10.1186/s12989-017-0207-3 (PMC5512939; doi:10.1186/s12989-017-0207-3)

Additional File 2

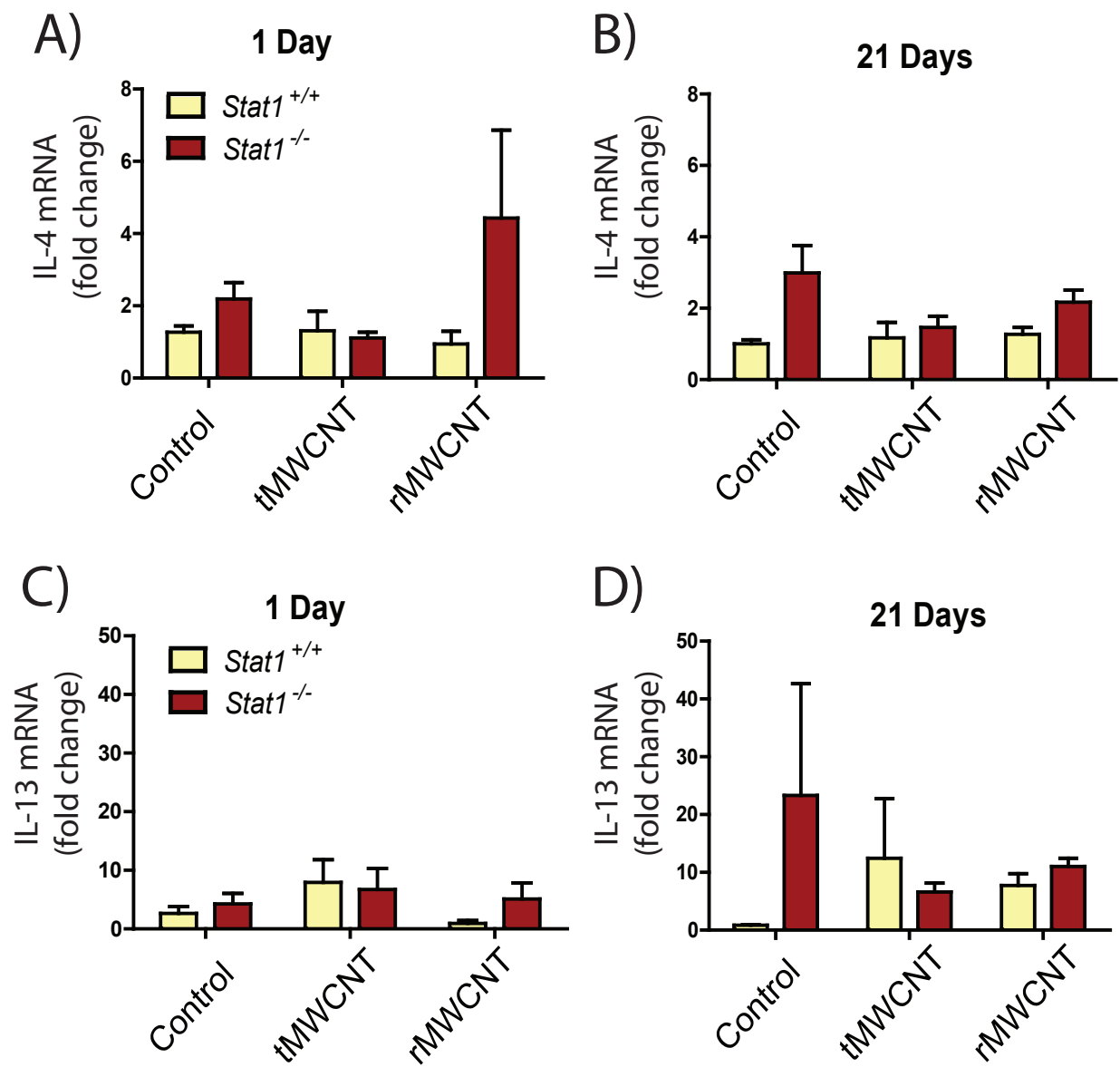

Supplement: Supplementary file 2 — Interleukin-4 (IL-4) and IL-13 mRNA expression in Stat1 +/+ and Stat1 −/− mouse lungs after exposure to tMWCNTs or rMWCNTs. A) Fold change in IL-4 mRNA at one and B) 21 days post-exposure. C) Fold change in IL-13 mRNA expression at one and D) 21 days post-exposure. Expression of mRNA normalized to β2-microglobulin (B2M). (PDF 352 kb) [file 12989_2017_207_MOESM2_ESM.pdf]

# Additional File 3

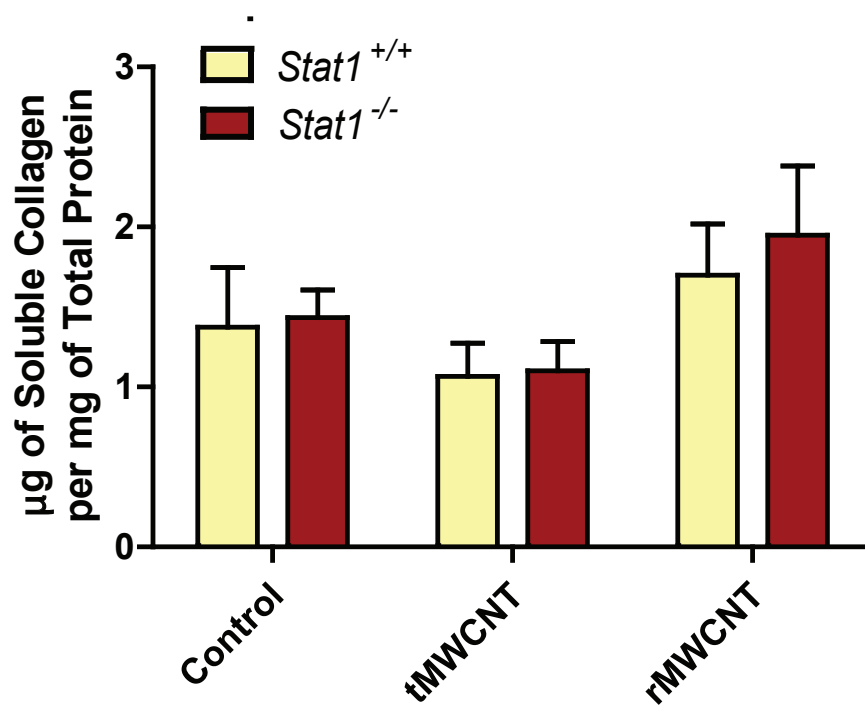

Supplement: Supplementary file 3 — Soluble collagen content measured from mouse lungs 21 days post-exposure. Average soluble collagen concentration per lung in each respective treatment after 21 days of exposure to control, tMWCNTs, or rMWCNTs normalized to protein content of sample. (PDF 314 kb) [file 12989_2017_207_MOESM3_ESM.pdf]

Additional File 4

A)

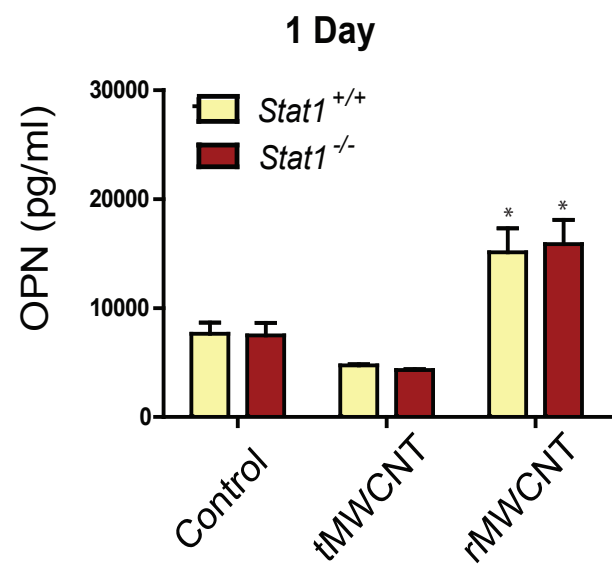

B)

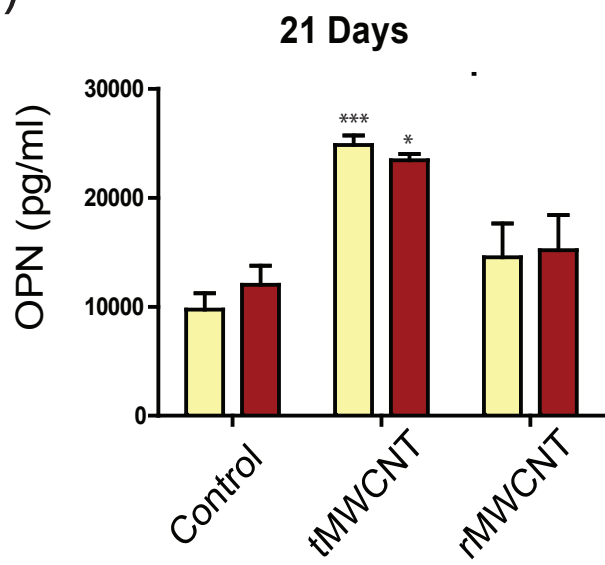

Supplement: Supplementary file 4 — Osteopontin (OPN) protein levels in lungs from Stat1 +/+ and Stat1 −/− mice after one and 21 days of exposure to tMWCNTs or rMWCNTs. A) OPN protein in BALF after one and B) 21 days exposure to vehicle, tMWCNTs, or rMWCNTs as measured by ELISA. (*p < 0.05 or ***p < 0.001 compared to control). (PDF 329 kb) [file 12989_2017_207_MOESM4_ESM.pdf]

A)

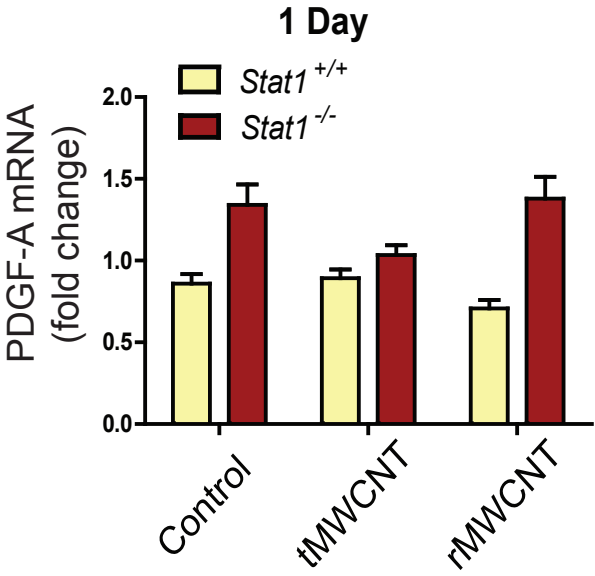

B)

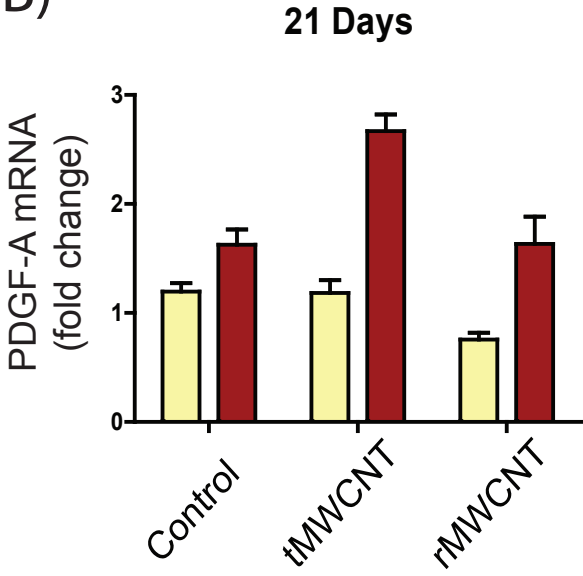

C)

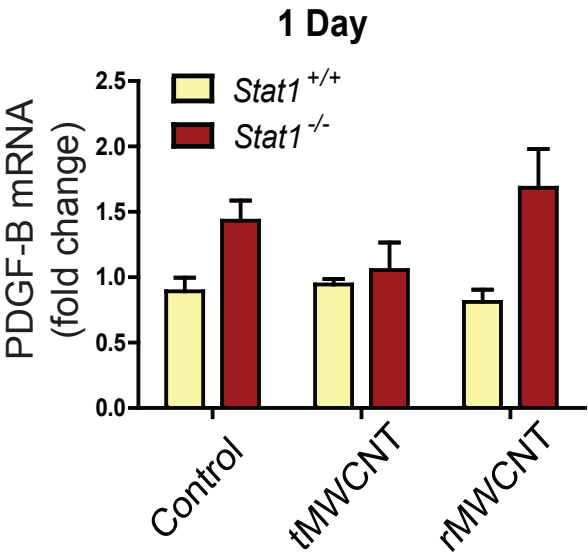

D)

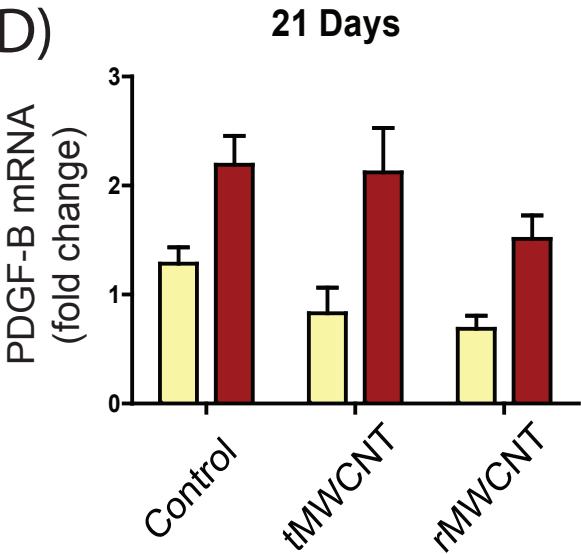

Supplement: Supplementary file 5 — Platelet derived growth factor (PDGF) -A and -B expression in Stat1 +/+ and Stat1 −/− mouse lungs after exposure to tMWCNTs or rMWCNTs. A) Fold change in PDGF-A mRNA at one and B) 21 days post-exposure. C) Fold change in PDGF-B mRNA expression at one and D) 21 days post-exposure. Expression of mRNA normalized to B2M. (PDF 356 kb) [file 12989_2017_207_MOESM5_ESM.pdf]

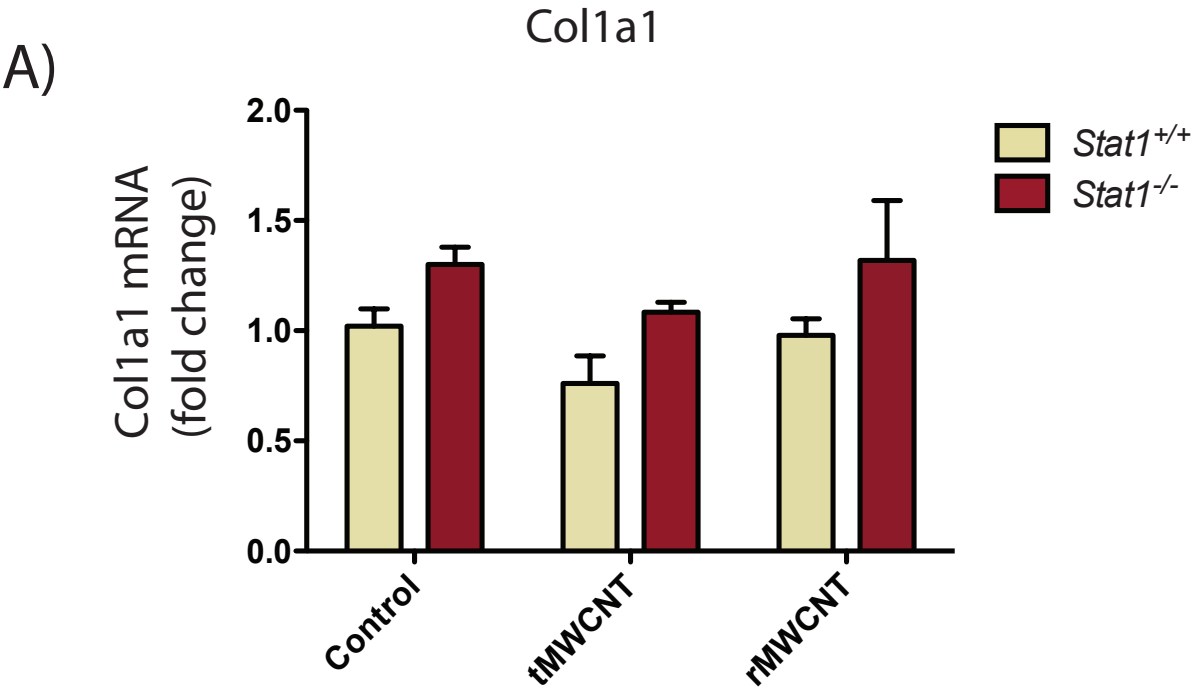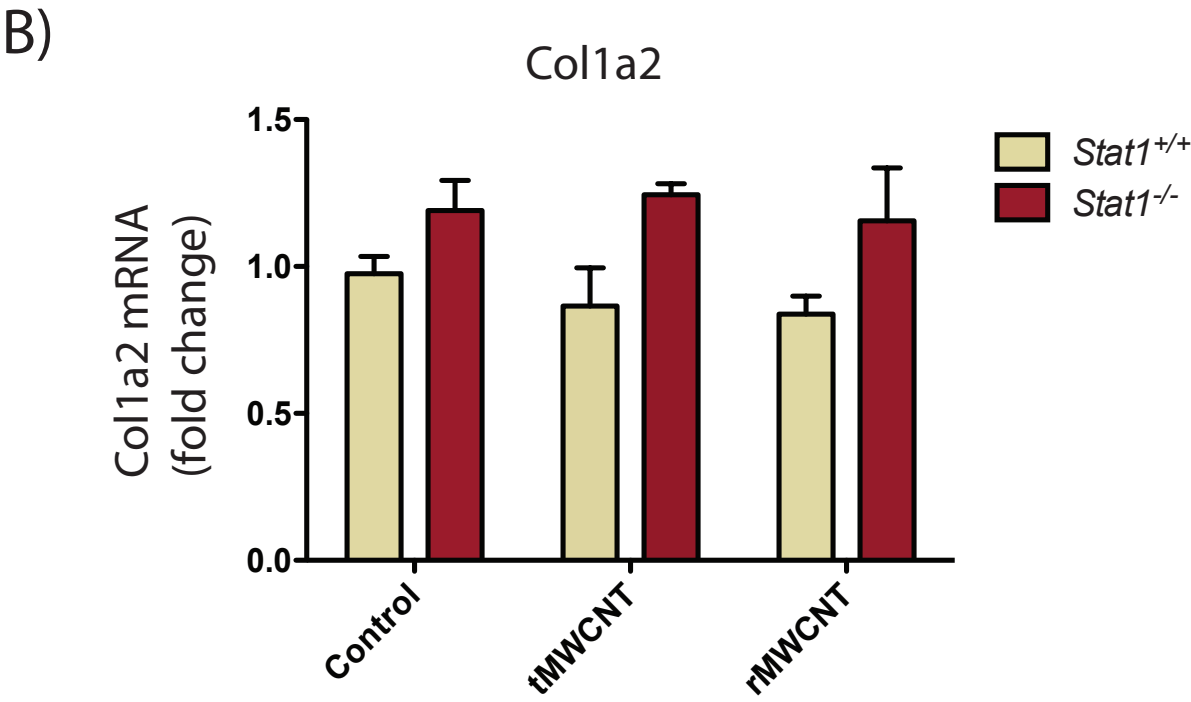

Supplement: Supplementary file 6 — Expression levels of collagen mRNAs determined via Taqman qRT-PCR of RNA isolated from mouse lungs 21 days post-exposure. A) Fold change in Col1a1 and B) Col1a2 mRNA expression after 21 days post-exposure. Expression of mRNA levels normalized to B2M. (PDF 137 kb) [file 12989_2017_207_MOESM6_ESM.pdf]
